# Long Term Culture of the A549 Cancer Cell Line Promotes Multilamellar Body Formation and Differentiation towards an Alveolar Type II Pneumocyte Phenotype

**DOI:** 10.1371/journal.pone.0164438

**Published:** 2016-10-28

**Authors:** James Ross Cooper, Muhammad Bilal Abdullatif, Edward C. Burnett, Karen E. Kempsell, Franco Conforti, Howard Tolley, Jane E. Collins, Donna E. Davies

**Affiliations:** 1 Public Health England, Culture Collections, Porton Down, Salisbury, Wiltshire, United Kingdom; 2 Academic Unit of Clinical and Experimental Sciences, Sir Henry Wellcome Laboratories, University of Southampton Faculty of Medicine, University Hospital Southampton, United Kingdom; 3 Public Health England, Diagnostic Technologies, Porton Down, Salisbury, Wiltshire, United Kingdom; 4 National Institute for Health Research, Respiratory Biomedical Research Unit, University Hospital Southampton, Southampton, United Kingdom; 5 Public Health England, Microbiology Services, Porton Down, Salisbury, Wiltshire, United Kingdom; University of Palermo, ITALY

## Abstract

Pulmonary research requires models that represent the physiology of alveolar epithelium but concerns with reproducibility, consistency and the technical and ethical challenges of using primary or stem cells has resulted in widespread use of continuous cancer or other immortalized cell lines. The A549 ‘alveolar’ cell line has been available for over four decades but there is an inconsistent view as to its suitability as an appropriate model for primary alveolar type II (ATII) cells. Since most work with A549 cells involves short term culture of proliferating cells, we postulated that culture conditions that reduced proliferation of the cancer cells would promote a more differentiated ATII cell phenotype. We examined A549 cell growth in different media over long term culture and then used microarray analysis to investigate temporal regulation of pathways involved in cell cycle and ATII differentiation; we also made comparisons with gene expression in freshly isolated human ATII cells. Analyses indicated that long term culture in Ham’s F12 resulted in substantial modulation of cell cycle genes to result in a quiescent population of cells with significant up-regulation of autophagic, differentiation and lipidogenic pathways. There were also increased numbers of up- and down-regulated genes shared with primary cells suggesting adoption of ATII characteristics and multilamellar body (MLB) development. Subsequent Oil Red-O staining and Transmission Electron Microscopy confirmed MLB expression in the differentiated A549 cells. This work defines a set of conditions for promoting ATII differentiation characteristics in A549 cells that may be advantageous for studies with this cell line.

## Introduction

Alveolar Type 1 (ATI) and 2 (ATII) cells are specialised epithelial cells of the distal lung. ATI cells are flattened squamous cells that cover around 95% of the alveolar surface and lie adjacent to capillary endothelial cells to form the pulmonary gas exchange region. ATII cells have a compact morphology and cover the remaining 5% of the alveolar surface. Unlike terminally differentiated and-non replicative ATI cells, ATII cells have multiple roles and have been described as the ‘defenders of the alveolus’[1,2]. The ultrastructural hallmark of ATII cells is the expression of multilamellar bodies (MLB)[3] containing dipalmitoylphosphatidyl choline (DPCC), the major lipid component of pulmonary surfactant that reduces surface tension in the alveoli to prevent collapse of the lungs at the end of expiration. ATII cells play an important role in innate immune responses within the lung with evidence that lung surfactant proteins have anti-microbial effects and reduce inflammation caused by the inhalation of irritants. ATII cells also help clear alveolar fluid through active sodium transport and they act as self-renewing progenitors to replace ATI cells that have been damaged[4] to maintain normal lung architecture[5–7].

Research into alveolar physiology and pathologies relevant to acute lung injury[8,9], and diseases such as chronic obstructive pulmonary disease (COPD)[10,11] and interstitial lung diseases such as idiopathic pulmonary fibrosis[12–15] requires *in vitro* models that represent and mimic the alveolar epithelium, in particular the ATII cell. Primary ATII cell cultures are currently considered to be the most useful *in vitro* model for alveolar research, however they are limited by tissue availability which requires ethical approval and patient consent for access to histologically normal regions of resected lung tissue surplus to requirement for diagnosis of lung carcinoma [16,17]. While these cells are useful in short term culture, they spontaneously differentiate to the ATI phenotype over 1–2 weeks[18]. Recent developments have promised the potential of alveolar models from human embryonic stem cells[19], mesenchymal stem cells[20] and induced pluripotent stem cells[21,22], however technical difficulties and issues presented by these systems have limited their widespread uptake and use. As a consequence, there is still considerable reliance and widespread use of authentic[23] continuous cancer or other immortalized cell lines. Sometimes these cell lines are derived by retroviral transduction, as has been demonstrated with mammary and endothelial tissues[24], but more commonly they have been derived from tumours—often many decades previously. These continuous cell lines have the major advantage of ease of cultivation, reproducibility and relatively unlimited supply. However, although they can maintain a stable phenotype through many subcultures if properly maintained[25], this phenotype exhibits differences compared to the original tissue, compromising their ability to fully reproduce *in vivo* physiological state. Often their use is a trade-off of ‘ease of use’ against suitability, as the cells typically retain features more associated to the original tumour, including uncontrolled proliferative growth and a de-differentiated phenotype. One such commonly used cell model is the lung carcinoma cell line A549. Isolated in 1973 from a pulmonary adenocarcinoma[26] and subsequently characterized as being representative of ATII cells[3,27–29], this cell line has been a mainstay of respiratory research for nearly four decades. However while work with early passage A549 cells provided evidence of their ability to exhibit features of an ATII epithelial cell phenotype[27–29], more recent studies have led to conflicting results[30,31]. Based on early work with A549 cells which reported that extended culture resulted in cellular ‘differentiation’, as evidenced by high numbers of MLB[3,32], we tested the hypothesis that culture conditions that reduce proliferation of the A549 cell line would promote a more differentiated ATII cell phenotype, as evidenced by mRNA gene expression profiling over time, by comparison with primary cultures of ATII cells and by histological and ultrastructural analysis.

## Materials and Methods

### Cell Culture

Authentic A549 cells (European Collection of Cell Cultures (ECACC), Salisbury, UK), catalogue number 86012804, were cultured in either Ham’s F12 Nutrient Medium (Ham’s F12) or Dulbecco’s Modified Eagles Medium (DMEM) (both from Sigma Aldrich, Dorset, United Kingdom) supplemented with 2mM L-Glutamine and 10% v/v Foetal Bovine Serum (FBS) (Hyclone SH30071.03 (Hyclone Laboratories, Utah, USA). Proliferative cultures were incubated at 37°C in a humidified 5% CO_2_ incubator and subculture carried out by washing the cell monolayers twice with calcium and magnesium-free phosphate buffered saline (PBS) (Severn Biotech (Kidderminster, UK, catalogue number 20–74) followed by addition of 1x Trypsin/EDTA solution (Sigma Aldrich) and incubation at 37°C until the cells detached. Trypsin was inactivated by the addition of growth medium before seeding into fresh flasks at densities of 1.5-2x10^4^ cells/cm^2^. For the long term 25 day cultures A549 cells were seeded into replicate T25 flasks and medium changed every 2–4 days. Phase contrast images were captured of the monolayers throughout the time course. Cell numbers, viability and size were assessed by Trypan Blue staining and by DAPI dye exclusion using the Nucleocounter^™^ 3000 viability assay (Chemometec, Allerod, Denmark).

### RNA Extraction

Cells were harvested using trypsin/EDTA, counted and washed with PBS by centrifugation at 500g for 5 minutes and snap frozen in pellets of 1.5 x 10^6^ cells before storage at -80°C prior to RNA extraction. RNA was extracted using the Promega Maxwell^®^ Low Elution Volume (LEV) Simply RNA Cell kit according to the manufacturer’s instructions. In brief 16 samples were processed at a time. Pellets were thawed rapidly and as briefly as possible in a 37°C water-bath, transferred to wet ice where 200μl of homogenisation solution was added to each sample and vortexed to mix before the addition of 200 μl of lysis solution and another vortex mix prior to loading into the Maxwell^®^ cassettes. DNAse was added to remove contaminating genomic DNA. RNA was eluted into 50μl of nuclease free water and supplemented with RNAse inhibitor (“Superase In^™^”, Ambion, Life Technologies, Paisley, UK) before being quantified by spectrophotometry (Nanodrop, Labtech International, Uckfield East Sussex UK) and analyzed by electrophoresis in a 1.4% agarose (Sigma Aldrich) gel and visualised using Ethidium Bromide and UV illumination to ensure there were intact 18S and 28S bands. Extracted RNA was stored at -80°C before RNA microarray analysis prior to which repeat assessment of RNA integrity was carried out using a bio-analyzer (BioAnalyzer 2100, Agilent Technologies, Santa Clara, USA).

### Primary ATII Cells

Primary ATII cells were isolated by protease digestion and selective adherence according to published protocols[16] and the commonly adopted strategy of using macroscopically normal tissue from three ex-smokers undergoing lung resection: Donor 1 (female, aged 57), Donor 2 (female, aged 69) and Donor 3 (male, aged 69). Written consent from the donors of the primary lung tissue was given under the governance of the National Health Service (NHS) England Southampton and South West Hampshire ‘A’ Research Ethics Committee. Local Research Ethics Committee (LREC) Reference Number 08/H0502/32.

The purified ATII cells were re-suspended in DCCM-1 medium (Biological Industries, Israel) supplemented with 1% penicillin, 1%streptomycin and 1% L-glutamine and 10% NCS before plating on collagen I (PureCol 5005-b, Advanced BioMatrix Inc, Carlsbad, USA) coated 24 well plates at 60% confluence. The presence of ATII cells was confirmed by staining for alkaline phosphatase. Replicate RNA samples were isolated from wells of the 24 well plate using Trizol according to the manufacturer’s instructions (Life Technologies, Paisley, UK).

#### QRT-PCR

The extracted RNA was quantified (Denovix, Wilmington, Delaware, USA) and 3ng of each of the RNA samples was then reverse transcribed to cDNA using the Superscript ^®^ II Reverse Transcriptase Kit (Oligo dt) (Life Technologies, Paisley, UK) to the manufacturer’s instructions.

QRT PCR was performed on the cDNA using a Quant-Studio 7 thermocycler (Life Technologies, Paisley, UK), and curated Taqman assays (SFTPA1 (Taqman assay ID Hs00831305_m1), SFTPA2 (Taqman assay ID Hs00359837_m1), SFTPB (Taqman assay ID Hs01090667_m1), SFTPC (Taqman assay ID Hs00161628_m1), SFTPD (Taqman assay ID Hs01108490_m1) (Life Technologies, Paisley, UK) using delta-delta Ct analysis to determine relative gene expression[33]. Ct values were normalised to the geometric means of those obtained from the reference genes topoisomerase (TOP1) (Taqman assay ID Hs00243257_m1) and ATP synthase subunit beta, mitochondrial (ATP5B) (Taqman assay ID Hs 00969569_m1) based on the results of a ‘genorm’ analysis[34] to determine the optimal reference genes. cDNA from log phase A549 cultures was used as the baseline for comparison of relative gene expression for all surfactant proteins except SFTPA2, where cDNA from 25 day differentiated A549 was used.

### RNA Microarray Analysis

cRNA labelled with Cyanine 3 was generated from the extracted RNA samples using the Agilent Single Color Low Input Quick Amp Labelling kit and purified prior to hybridisation to Agilent Human Single Color 39494 array slides. Genespring version 13 (Agilent Technologies, Santa Clara, USA). Data was quality controlled by excluding any compromised entities and only entities where all replicates were either detected or not detected. Samples were normalised by shift to the 75th percentile and the baseline transformation on the median of all samples and statistical and comparative analysis at the probe and gene level using parametric statistical analyses including analysis of variance (one way ANOVA) with Benjamini and Hochberg False Discovery Rate (BH FDR) correction. Pathway analyses using Genespring and Wiki Pathways[35–37] were carried out on genes up or down regulated at a fold change of two or more and on the genes shared with differentiated A549 and the ATII cells.

### Oil Red O staining

Cell monolayers were rinsed with phosphate buffered saline (PBS), fixed with two applications of 10% formalin before rinsing with purified water followed by addition of 60% isopropanol and air drying. The cells were stained using Oil red-O (Sigma Aldrich, Dorset, UK) (0.21g/ml in isopropanol, filtered), rinsed four times in purified water before imaging by light microscopy.

### Transmission Electron Microscopy

Cells were fixed in 2.5% glutaraldehyde solution before staining in osmium tetroxide and embedding in Araldite resin. The resulting blocks were sectioned, placed onto grids before further staining with 2% uranyl acetate and 0.1% lead citrate. Sections were imaged by transmission electron microscopy using Philips CM100 (Philips Electron Optics, Cambridge, UK) and Hitachi H7000 instruments (Hitachi Group, Maidenhead, Berkshire, UK).

To determine the proportion of cells expressing MLBs, 164 cells were assessed by direct counting of TEM micrographs.

## Results

**Fig 1:**
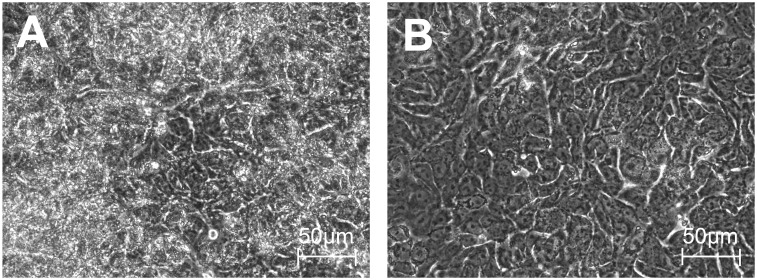
Phase contrast images of A549 monolayers. Images show the differences in morphology of 25 day continuous culture in DMEM (A) or Ham’s F12 (B).

**Fig 2:**
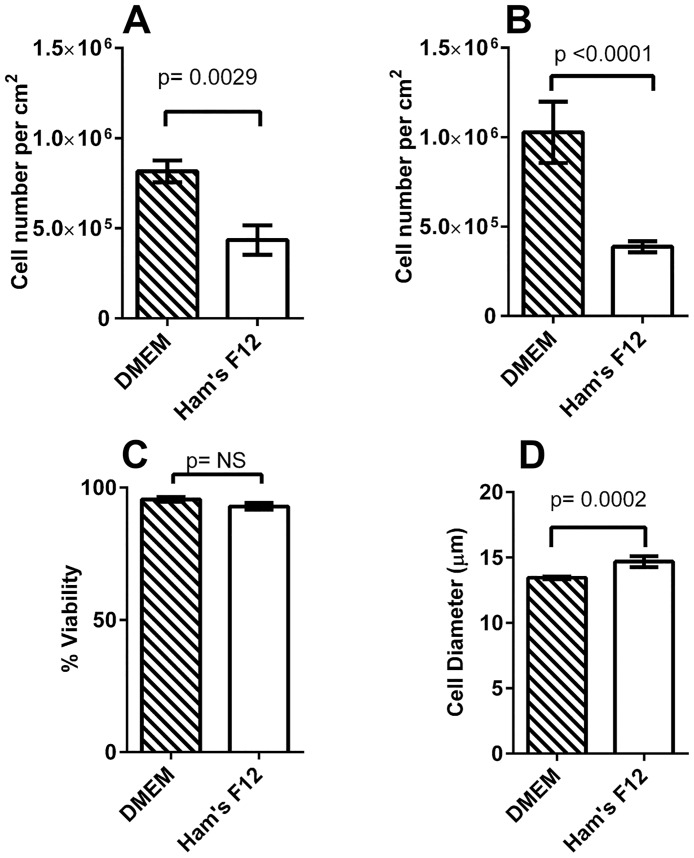
Cell metrics. Cell counts, viability and cell diameters from A549 cell cultures at day 25. A: cell yield per unit area based on cell counting using Trypan blue dye exclusion (n = 3) and B: cell numbers as measured by automated cell counting (n = 5). C: Cell viability based on DAPI dye exclusion (n = 5). D: cell diameter using automated image analysis (n = 5). Data are plotted as mean ± SD; statistical analyses in A, B and D used unpaired Student’s T Test. In C statistical testing showed no significant difference (NS).

It is not clear from the literature which media might induce the most ATII—like phenotype in cultured A549 cells. We compared the effects of two media: Ham’s F12 and DMEM on cell proliferation. After 24 hours of plating, the cells cultured in either medium appeared morphologically similar with mitotic cells evident in both conditions. Cells continued to divide as the cultures progressed, however there appeared to be more cell division, crowding and piling up of cells in DMEM (Fig 1A). In contrast, in Ham’s F12, the cells displayed a more flattened contact-inhibited quiescent appearance that was maintained until the cultures were terminated at day 25 (B). At this point, cell counts determined using two independent methods demonstrated that culture of cells in DMEM yielded significantly more cells than Ham’s F12 (Fig 2A and 2B). Although there was no difference in cell viability between the two culture conditions, cells grown in DMEM had a significantly smaller diameter (Fig 2C and 2D). A series of photomicrographs comparing the growth and morphology over the time course is included in S1 Fig. Closer examination of the cells cultured in Ham’s F12 showed the presence of organized vesicles of uniform size within the cells (Fig 1B and S1H Fig (inset)) suggesting the possibility of cellular differentiation.

**Fig 3:**
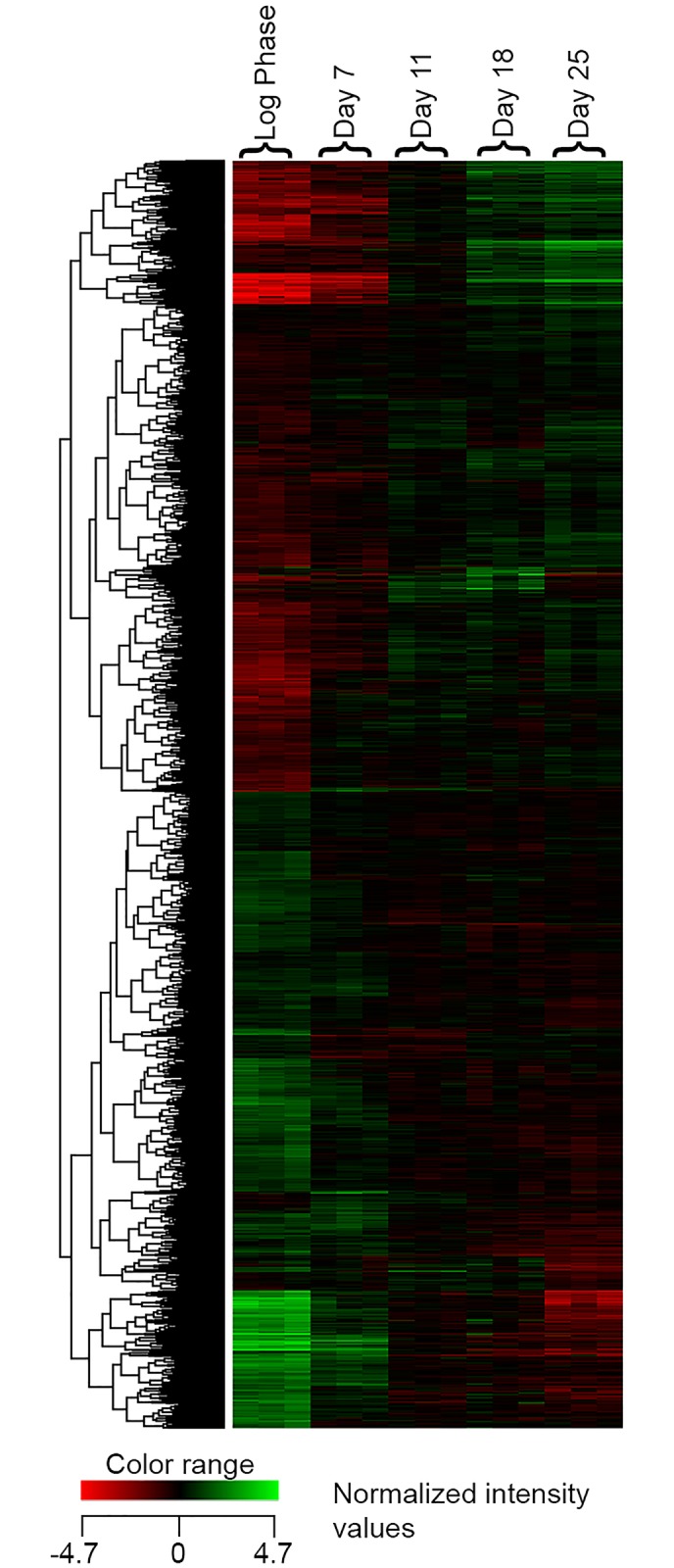
RNA Microarray Heatmap of Gene Expression. Hierarchically clustered (Euclidian similarity measure, clustered using Ward’s linkage rule) heat map of gene expression changes in A549 cells cultured in Ham’s F12 for up to 25 days; the heat map shows normalised intensity values of significant genes regulated up or down two-fold or more.

To obtain deeper insight into the changes occurring in A549 cells during long term culture in Hams F12, we used RNA microarray analysis to evaluate how gene expression changed in comparison with log phase cells. BH FDR corrected ANOVA analysis of microarray data demonstrated that of the 39,013 genes examined, 5,346 were significantly up or down regulated (p<0.05) during the 25 day time course and, of these, 3,926 were up or down regulated with a fold change of two or greater (Fig 3). Pathway analysis of the genes regulated at a fold change of two or more highlighted nineteen pathways with significant p values involved with aspects of cell cycle regulation (Table 1). Within these pathways, the markers of proliferation KI-67, PCNA and TCF7L1 were down regulated and the inhibitor of cell cycle progression CDKN1B was upregulated during the 25 day time-course (see S2 Fig). These findings are consistent with reduced proliferation in the cultures over 25 days of culture in Ham’s F12.

**Table 1:** Pathways Associated with Cell Cycle Control.

Pathway	Wiki-Pathway Reference	P Value	Number of regulated genes	Number of genes in pathway
Cell Cycle	WP179 70629	<0.001	51	103
S Phase	WP2772 77049	<0.001	46	116
Mitotic G1-G1-S phases	WP1858 76928	<0.001	46	120
Mitotic Metaphase and Anaphase	WP2757 77009	<0.001	57	153
Synthesis of DNA	WP1925 76968	<0.001	39	94
Nuclear Receptors Meta-Pathway	WP2882 78569	<0.001	80	318
RB in Cancer	WP2446 78573	<0.001	55	87
Mitotic Prometaphase	WP2652 76819	<0.001	54	98
DNA Replication	WP466 76196	<0.001	31	42
G1 to S cell cycle control	WP45 71377	<0.001	34	68
Telomere Maintenance	WP1928 76893	<0.001	24	37
Cell Cycle Checkpoints	WP1775 76816	<0.001	38	115
Nucleosome assembly	WP1874 76826	<0.001	15	22
miRNA Regulation of DNA Damage Response	WP1530 78503	<0.001	28	98
DNA Damage Response	WP707 78527	<0.001	27	68
Mitotic G2-G2-M phases	WP1859 77022	<0.001	31	89
M-G1 Transition	WP2785 77074	<0.001	27	79
Regulation of DNA replication	WP1898 76824	<0.001	18	70
Mitotic Prophase	WP2654 76823	0.002	12	44

Statistically significant pathways associated with cell cycle contrl for genes expressed over the 25 day time-course of A549 differentation in Ham’s F12 medium. Pathways were identified by Genespring pathway analysis after one way ANOVA of all of time points compared to log-phase A549 cells (P cut off = 0.05, Fold change ≥ 2.0).

In addition to a reduction in expression of genes involved in cell proliferation, a small number of pathways involved in cellular autophagy, senescence and apoptosis were modulated during long term culture (Tables 2 and 3). In contrast, there were 28 pathways related to differentiation of epithelial and endodermal tissues (Table 4). For example, WNT4, which has been implicated in lung development and repair, was upregulated as were the pluripotency markers Nanog and SOX2. Parallel up-regulation of SOX9 was also observed along with the WNT target Metalloproteinase 7 (MMP7) (see S3 Fig). A feature suggestive of ATII cell differentiation was significant expression of complement component pathways with, for example, increased expression of C3, C4b and C5 (S4 Fig). In addition to induction of differentiation pathways, Table 5 shows 10 pathways involved in lipid metabolism that achieved statistical significance over the 25 day time course.

**Table 2:** Pathways Associated with Apoptosis.

Pathway	Wiki-Pathway Reference	P Value	Number of regulated genes	Number of genes in pathway
Apoptosis	WP254 78808	0.021	17	84
FAS pathway and Stress induction of HSP regulation	WP314 71366	0.033	9	38
Apoptotic execution phase	WP1784 76813	0.033	10	46

Statistically significant pathways associated with apoptosis for genes expressed over the 25 day time-course of A549 differentiation in Ham’s F12 medium. Pathways were identified using Genespring pathway analysis after one way ANOVA of all of the time points compared to log-phase A549 cells (P cut off = 0.05, Fold change ≥ 2.0).

**Table 3:** Pathways Associated with Senescence and Autophagy.

Pathway	Wiki-Pathway Reference	P Value	Number of regulated genes	Number of genes in pathway
Senescence and Autophagy	WP615 71375	<0.001	32	106
AMPK Signaling	WP1403 78804	0.006	16	68
Folate Metabolism	WP176 74202	0.020	14	67

Statistically significant pathways associated with senescence and autophagy for genes expressed over the 25 day time-course of A549 differentiation in Ham’s F12 medium. Pathways were identified using Genespring pathway analysis after one way ANOVA of all of the time points compared to log-phase A549 cells (P cut off = 0.05, Fold change ≥ 2.0).

**Table 4:** Pathways Associated with Epithelial and Endodermal Differentiation.

Pathway	Wiki-Pathway Reference	P Value	Number of regulated genes	Number of genes in pathway
miR-targeted genes in epithelium—TarBase	WP2002 78530	<0.001	69	345
Complement and Coagulation Cascades	WP558 67786	<0.001	21	64
TGF Beta Signaling Pathway	WP366 69026	<0.001	21	55
TGF Beta Signaling Pathway	WP560 68944	<0.001	21	55
Cell junction organization	WP1793 77057	<0.001	20	61
Human Complement System	WP2806 78589	<0.001	28	136
Signaling by TGF-beta Receptor Complex	WP2742 76980	<0.001	13	36
Focal Adhesion	WP306 78800	<0.001	41	188
Integrin cell surface interactions	WP1833 77019	<0.001	19	64
Oncostatin M Signaling Pathway	WP2374 73668	<0.001	19	65
Integrin-mediated Cell Adhesion	WP185 71391	<0.001	24	99
Complement cascade	WP1798 77042	<0.001	13	192
Assembly of collagen fibrils and other multimeric structures	WP2798 77089	8.62E-04	11	33
Endoderm Differentiation	WP2853 78496	<0.001	18	146
Differentiation Pathway	WP2848 78558	0.001	14	50
Wnt Signaling Pathway and Pluripotency	WP399 74897	0.003	22	100
Cell Differentiation—meta	WP2023 68892	0.005	7	67
ID signaling pathway	WP53 67360	0.006	6	16
Prostaglandin Synthesis and Regulation	WP98 72088	0.008	9	31
Cell surface interactions at the vascular wall	WP1794 77039	0.008	19	91
Constitutive Androstane Receptor Pathway	WP2875 78537	0.009	8	32
EGF-EGFR Signaling Pathway	WP437 78502	0.010	30	162
Complement Activation, Classical Pathway	WP545 72062	0.011	6	17
Angiogenesis	WP1539 78807	0.015	7	24
Mitotic Telophase-Cytokinesis	WP2765 77030	0.020	5	14
Activation of Matrix Metalloproteinases	WP2769 77041	0.035	5	16
Gap junction trafficking and regulation	WP1820 76886	0.042	3	8
Wnt Signaling Pathway	WP428 78532	0.046	12	61

Statistically significant pathways associated with epithelial and endodermal differentiation for genes expressed over the 25 day time-course of A549 differentiation in Ham’s F12 medium. Pathways were identified by Genespring pathway analysis after one way ANOVA of all of the time points compared to log-phase A549 cells (P cut off = 0.05, Fold change ≥2.0).

**Table 5:** Pathways Associated with Lipid Synthesis and Metabolism.

Pathway	Wiki-Pathway Reference	P Value	Number of regulated genes	Number of genes in pathway
Adipogenesis	WP236 78584	<0.001	38	131
SREBF and miR33 in cholesterol and lipid homeostasis	WP2011 75253	<0.001	9	18
Nuclear Receptors in Lipid Metabolism and Toxicity	WP299 78587	<0.001	12	35
Ganglio Sphingolipid Metabolism	WP1423 76323	0.002	7	18
Signal Transduction of S1P Receptor	WP26 78492	0.005	8	25
Cholesterol biosynthesis	WP1795 77044	0.020	6	21
Regulation of Lipid Metabolism by Peroxisome proliferator-activated receptor alpha (PPARalpha)	WP2797 77088	0.025	21	118
Globo Sphingolipid Metabolism	WP1424 71392	0.026	6	24
Cholesterol Biosynthesis	WP197 78758	0.027	5	15
Fatty Acid Biosynthesis	WP357 70641	0.041	6	22

Statistically significant pathways associated with lipid synthesis and metabolism for genes expressed over the 25 day time-course of A549 differentiation in Ham’s F12 medium. Pathways were identified using Genespring pathway analysis after one way ANOVA of all of the time points compared to log-phase A549 cells (P cut off = 0.05, Fold change ≥ 2.0).

To determine whether modulation of A549 cell growth and differentiation resulted in their transition towards an ATII cell phenotype, we compared gene expression of the long term cultured A549 cells to that of freshly isolated human primary ATII cultures. Comparison of relative gene expression using primary ATII cells from three donors showed a similar pattern of expression of five surfactant proteins (SFTPD, A2, A1, B and C) (S5 Fig); therefore we selected Donor 2 for RNA microarray studies to represent normal ATII cells. In this analysis each of the time-points of A549 differentiation and the primary ATII cells was compared to log phase A549 cells as the point at which it is assumed that most researchers would use their A549 cells in experiments.

**Fig 4:**
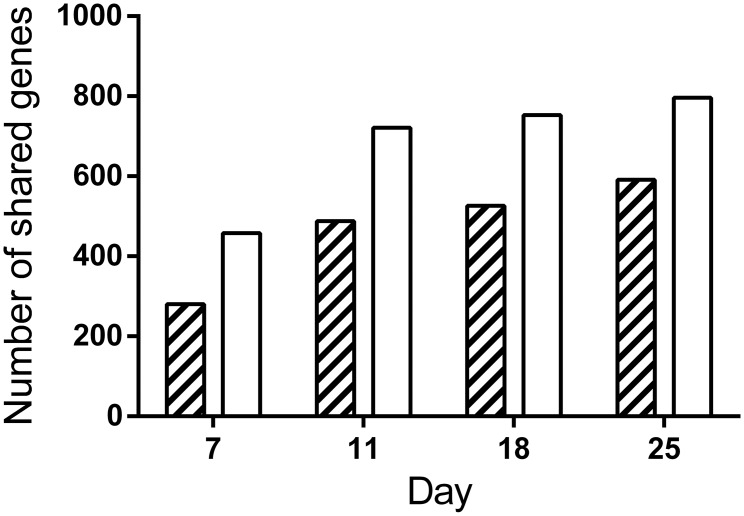
Shared up regulated (≥ 2 fold) gene expression of differentiated A549 with freshly isolated human primary ATII cultures. Gene expression in A549 cells that were cultured for 7, 11, 18 or 25 days or primary ATII cells were compared to log phase A549 cells. The figure shows the number of shared up- (hatched bars) or down- (clear bars) regulated genes between the A549 cells and ATII cells over the A549 time course.

The RNA microarray comparison of primary ATII cells with long term cultured A549 showed that for a minimum of fold change of two, the number of shared up regulated genes increased from 280 at day 7 of the differentiation time-course to 591 at day 25. Similarly the number shared down regulated genes increased from 458 at day 7 to 796 at day 25 (Fig 4). Analysis of the shared up regulated genes (Table 6) indicated pathways involved with the Complement System (C3 and C4b), senescence and autophagy, lipid metabolism (including fatty acid biosynthesis, adipogenesis, sphingolipid metabolism, cholesterol and lipid homeostasis, peroxisome proliferator activated receptor alpha), endodermal and cellular differentiation and TGF beta signalling.

**Table 6:** Pathways Considered to be Associated with ATII Phenotype.

Pathway	Wiki Pathways Reference	Most significant timepoint (Day)	P Value	Number regulated genes	Number genes in pathway
Complement cascade	WP1798 77042	25	<0.001	9	192
Human Complement System	WP2806 78589	18	<0.001	12	136
Senescence and Autophagy	WP615 71375	25	<0.001	9	106
Complement Activation, Classical Pathway	WP545 72062	25	<0.001	4	17
Complement and Coagulation Cascades	WP558 67786	18	<0.001	6	64
Adipogenesis	WP236 78584	11	<0.001	10	131
Differentiation Pathway	WP2848 78558	11	0.001	5	50
Globo Sphingolipid Metabolism	WP1424 71392	18	0.005	3	24
Sphingolipid Metabolism	WP1422 78591	25	0.006	3	21
Sphingolipid metabolism	WP2788 77079	25	0.010	4	46
SREBF and miR33 in cholesterol and lipid homeostasis	WP2011 75253	11	0.026	2	18
Ganglio Sphingolipid Metabolism	WP1423 76323	7	0.010	2	18
Regulation of Lipid Metabolism by Peroxisome proliferator-activated receptor alpha (PPARalpha)	WP2797 77088	11	0.009	6	118
TGF Beta Signaling Pathway	WP366 69026	7	0.013	3	55
TGF Beta Signaling Pathway	WP560 68944	7	0.013	3	55

Statistically significant pathways considered to be associated with ATII phenotype. Two fold (or greater) upregulated genes shared with primary ATII cells and differentiated A549 cells over the 25 day time-course identified by Genespring pathway analysis of Venn diagram analysis of the two cell populations.

Table 7 summarizes the fold changes in gene expression in a selection of key genes and markers involved in ATII differentiation in our experiments by comparing the relative fold change difference of differentiated A549 cells and primary ATII cells compared to log phase A549 cells. ATP lipid transporters have been associated with the organized transport of lipids into developing MLB and are considered a key marker of ATII cells. ABCA3 was expressed in abundance in the primary ATII cells but not significantly upregulated in the differentiated A549 cells. However, other lipid transporters were significantly upregulated in differentiated A549 cells (S6 Fig).

**Table 7:** Genes Related to ATII Differentiation.

Gene Symbol	Up or Down Regulation in A549 Cells throughout time-course	Fold Change at Day 25 in A549 Cells	Up or Down Regulation in ATII primaries compared to log phase A549	Fold Change (ATII vs log Phase A549)
Proliferation Markers				
Ki67	Down	-5.08	Down	-15.83
PCNA	Down	-2.19	Down	-6.17
TCF7L1	Down	-5.85	N/S	
Cell Cycle Inhibitor				
CDKN1B	Up	3.05	N/S	
Lipid Metabolism				
PPAPDC1B	Up	2.45	N/S	
PPAPDC1A	N/S		Up	15.19
PPAP2A	Up	2.62	Up	7.03
PPAP2B	Up	2.90	N/S	
DGAT2	Up	3.21	N/S	
FABP5	Down	-5.96	Down	-3.6
ACSL5	Up	16.00	Up	16.00
Autophagy and Lysosomal				
ULK4	Up	3.64	Down	-3.53
LAMP2	Up	2.08	Up	3.23
LAMP3	Up	2.65	Up	16.00
PLD1	Up	2.0	N/S	
PLD2	N/S		Up	2.83
PLD5	Up	4.97	N/S	
WNT Associated Differentiation				
CASP1	Up	16.00	Up	16.00
CASP4	Up	6.28	Up	16.00
BIRC5	Down	-16.00	Down	-16.00
WNT4	Up	12.10	Up	6.12
Stem Cell Markers and Differentiation				
NANOG	Up	4.06	Up	N/S
SOX2	Up	3.19	Up	4.75
SOX9	Up	2.79	N/S	
Complement Components				
C3	Up	14.92	Up	16.00
C4B	Up	12.00	Up	9.06
C4BPA	Up	16.00	Up	16.00
C5	Up	6.31	Down	-3.25
Cellular Differentiation				
IL1B	Up	16.00	Up	15.56
AGT	Up	16.00	Up	9.92
PPARA	Up	3.71	Up	4.16
FST	Up	8.81	Up	7.75
BMP4	Up	13.65	Up	7.98
TGFBR2	Up	2.93	Up	2.81
ATP Lipid Transporters				
ABCA3	N/S		Up	4.26
ABCC6P1	Up	5.07	Up	16.00
ABCC3	Up	2.7	Down	-8.11
ABCG1	Up	4.35	Down	-3.44
ABCA1	Up	2.48 (Day 7)	N/S	
ABCA8	Up	4.09	N/S	
ABCA12	Up	3.45	Up	5.06
ABCB4	Up	16.00	N/S	
ABCG2	Up	2.05	Down	-16.00
ABCD3	Down	-3.30	Down	-2.67
ABCC11	Up	4.45	N/S	
Matrix Metalloproteinases				
MMP1	Up	5.76	Up	16.00
MMP15	Down	-3.45	N/S	
MMP7	Up	6.65	Up	2.98
MMP9	Down	2.41	N/S	

Comparison of genes related to ATII differentiation in 25 day differentiated A549 cells grown in F12 compared to ATII Primary Cells compared to ATII Primary Cells using the gene expression of log phase proliferating A549 cells as a baseline.

**Fig 5:**
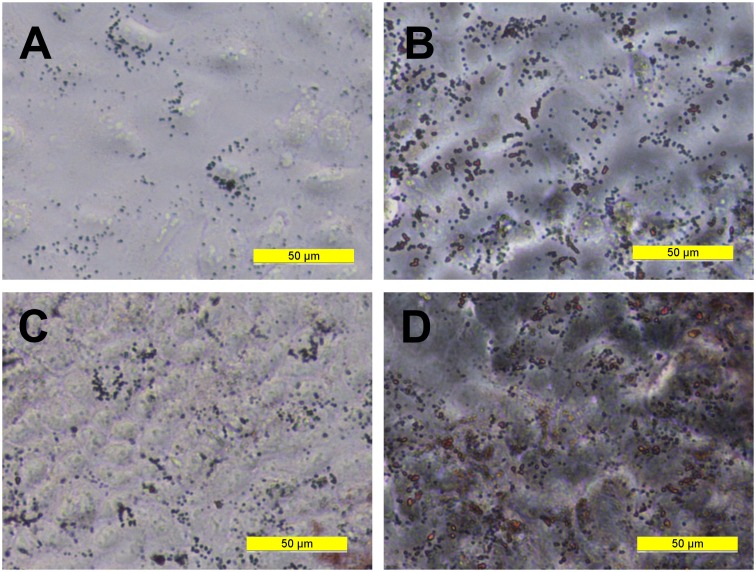
Oil-Red-O Staining of A549 Monolayers. Phase contrast images of Oil-Red-O staining of lipid droplets in log phase A549 monolayers grown in Ham’s F12 medium in log phase (A), and cells in the same medium for 7 (B), Day 11 (C) and Day 18 (D) days.

**Fig 6:**
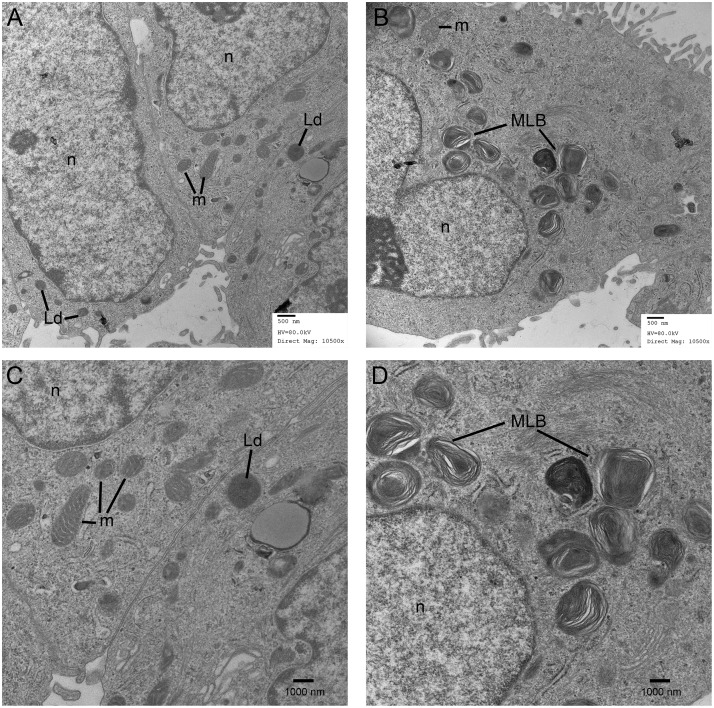
Electron Micrographs Showing Multilamellar Body Expression in A549 Cells. Transmission electron micrographs of sections of A549 cells after 11 (A and C) or 21 (B and B) days of culture in Ham’s F12. Lipid bodies (Lb), mitochondria (m), nuclei (n) and multilamellar bodies (MLB) are identified. Scale bars = 500 and 1000 nm.

To confirm the presence of lipidogenesis and possible MLB formation, differentiated A549 monolayers were stained with Oil-red-O. This showed that lipid production increased over the time course of differentiation (Fig 5A–5D) and was manifested by an increase in number and size of lipid inclusions, with more than half of the cells in the monolayer at day 18 showing evidence of lipid containing bodies. However from this staining it was not possible to discriminate MLB from oil droplets within the cells. Therefore, TEM microscopy was performed to assess the ultrastructure of the lipid droplets. At day 11, lipid droplets appeared as uniform structures with no evidence of MLB formation (Fig 6A and 6C). However by day 21 of differentiation, the lipid containing structures showed clear evidence of MLB formation (Fig 6B and 6D) with a distribution similar to that identified by the oil red-O staining. A differential count of 164 cells in TEM micrographs of long term differentiated A549 cells showed that 90 (54.9%) of the cells examined contained MLBs.

## Discussion

In this study, we report that long term culture of A549 cells in Ham’s F12 medium resulted in substantial suppression of genes involved in cell division in association with significant up-regulation of genes involved in autophagic, differentiation and lipidogenic pathways. There were also increased numbers of up- and down-regulated genes shared with primary ATII cells isolated using conventional methodology[31] suggesting adoption of some ATII characteristics including multilamellar body (MLB) development, a feature which was confirmed by electron microscopy. This work defines a set of conditions for promoting ATII differentiation characteristics in A549 cells that may be advantageous for functional studies with these cells. Novel alternative methods for ATII preparation have been defined from which future studies may benefit[38].

The evidence for the A549 cell line’s suitability as an *in vitro* ATII model is conflicting and is not fully explored in contemporary literature. The cell line was originally reported to have morphological and ultrastructural similarities to ATII cells. As long ago as 1978[3), Shapiro et al showed that by three weeks in continuous culture, cell division in A549 cells, as measured by DNA content, was low and the authors considered the cells as ‘differentiated’ as confirmed by the expression of high numbers of MLB similar in phospholipid content to those found in primary lung tissue. These findings were later supported by Nardone et al[29].

It is now well established that environmental factors such as choice of substratum, medium and continuous culture can have a substantial impact on the phenotype and gene expression. For example epithelial differentiation can be induced in continuous cell lines such as CaCo2[39] and MDCK[40] through the application of well-defined long term cell culture conditions. From the literature, it is evident that pulmonary researchers have not been consistent in the formulation of cell culture medium used in their experiments nor with the phase of growth from which the A549 cells are used. The cell line was originally isolated using RPMI medium[3] yet subsequent researchers have used a number of different media with little or no justification for their choice. By way of example, concerns for the cell line’s suitability were raised in 1980[41] with A549 cells cultivated in DMEM and subjected to a 10 day differentiation period. In this case the MLB content of the cells could be increased with 2 days of serum starvation, however, the lipid content of these A549 cells differed significantly from freshly isolated rat primary ATII cells and the authors advised that the cell line should be considered as a model of ATII dysfunction. More recently, using Raman spectroscopy to investigate the biochemical characteristics of A549 cells, Swain et al[30] cast further doubt on the A549 cell line’s performance as an ATII model but used cells grown in DMEM. Heijink et al[42] also reported functional deficiencies of the cell line using cells grown in RPMI 1640 medium. Based on our observations that DMEM supports a proliferative phenotype even in long term culture, it is possible that inconsistency in the choice of medium and/or culture duration used for A549 experiments may contribute to variability in phenotypic properties. This highlights the need for standardization in the use of A549 cells, and speaks to a larger problem that has been identified in life science research[43].

One of the key roles of ATII cells *in vivo* is to secrete surfactant, thus surfactant lipid production and evidence of MLB biogenesis in A549 cells, as originally reported[27] is supportive of their suitability as an ATII model. Lung surfactant has a role in immune protectiveness and the production of complement[44] and our finding of up-regulated gene expression of C3, C4b and C5 suggest the synthesis of components of the classical and alternate complement pathways by the long term A549 cell cultures consistent with their differentiation into a more ATII-like phenotype.

It is generally accepted that MLB biogenesis can be achieved by *de novo* synthesis of DPPC or alternatively through cellular autophagy[45,46]. Since MLB are thought to be lysosomal in origin, the expression of the Lysosomal Associated Membrane Proteins (LAMP) 2, and 3[46,47] and the lytic phospholipase enzymes PLD1 and 5[45] observed in the present study suggest that the long-term A549 cultures are developing lysosomes. Our data and pathway analyses further support the involvement of autophagy in MLB biogenesis with upregulation of autophagic pathways including the autophagy gene ULK4[48] in the long term A549 cultures. Autophagy seems to be an important process in the early development of the lung in particularly in the intervening period of starvation between birth and nutrient supply from maternal lactation. Autophagy provides a nutritional bridge at this critical stage at which point the lung has to adapt from an environment of amniotic fluid to breathing air and the immediate secretion of surfactant from MLBs[49]. Failure to respond in this manner can lead to infant respiratory distress syndrome[50] and mice with targeted deletions of individual autophagy genes such as ATG16L1[51,52] have high mortality rates in their offspring.

Lipid and fatty acid (FA) precursors for DPPC are not only derived from autophagy in the development of MLB, they can also be synthesized *de novo*. In adults it is thought that FAs are sequestered from the circulation via Fatty Acid Binding Proteins (FABP)[1], however our data show that FAB5 is down regulated throughout the A549 time course and in primary ATII cells, perhaps indicating that there are insufficient FAs provided in the culture medium or that the cells have switched to autophagic and biosynthetic generation of FAs. The significant upregulation of genes involved in lipid biosynthesis and metabolism support this hypothesis.

Membrane bound ATP lipid transporters, for example ABCA3[53], have been associated with the organized transport of lipids into developing MLB and are considered a key marker of ATII cells. Although ABCA3 was not significantly upregulated in the differentiated A549 cells, other ATP lipid transporters are involved MLB formation and surfactant production. For example ABCA1, 2, 3, and 5, have been implicated[54] and ABCA2 has been shown to be associated with the limiting membranes of MLB while other work has demonstrated that ABCA1 is enriched in the lung[55]. Our results with long-term cultures of A549 cells show the upregulation of several candidate ATP Lipid transporters that could play a part in MLB assembly.

Analysis of up-regulated genes that are shared between primary ATII cells and differentiated A549 confirmed that over the 25 day time-course of differentiation the A549 cell line became more similar in terms of gene expression to the ATII cells than log phase A549 cells. However while the 25 day differentiated A549 cultures are more similar to primary ATII cells there are still many differences. This may be because A549 cells retain an abnormal phenotype as a consequence of their malignant background, or because they consist of a phenotypically heterogeneous population possibly due to the presence of cancer stem cells with the potential to differentiate to ATII or non-ciliated bronchial cell types [32]. Increases in expression of the progenitor cell markers SOX2, SOX9 and NANOG in A549 cells seen in the present study could be indicative of the presence of such a cancer stem cell population[56].

As with all models, recapitulation of the *in vivo* state is imperfect but the aim is to reproduce, as faithfully as possible those aspects of the physiology (or pathology) that are being investigated. The gene expression data of the A549 time-course, the upregulated pathways and genes shared with primary ATII cells together with the confirmatory TEM data demonstrates that we have defined a reproducible and standard set of conditions for promoting ATII differentiation characteristics in A549 cells. In conclusion, we suggest that whereas proliferating log-phase A549 cells are most suitable for cancer biology studies, the new long term culture system would be more suitable for *in vitro* studies requiring a more representative and continuous source of ATII-like cells.

## Supporting Information

S1 Fig Images show the differences in morphology over 25 days of continuous culture in DMEM (top row, A-D) or Ham’s F12 (bottom row, E-H). Photomicrographs show morphology at day 1 (A and E), days 8 (B and F) and 14 (C and G) and day 25 (D and H). (Inset in H shows higher magnification of cells displaying organized vesicles in F12, inset in D shows a higher magnification of cells grown in DMEM for comparison).
(TIF)

S2 Fig Box and whisker plots of microarray RNA gene expression in A549 monolayers grown in Ham’s F12 (normalized intensity values) of proliferation markers Ki-67 (A), PCNA (B) and TCF7L1 (C) and cell cycle inhibitor CDKN1B (D) over the 25 day time course. ‘Day 0’ is representative of log phase A549 monolayers.
(TIF)

S3 Fig Box and whisker plots of microarray RNA gene expression in A549 monolayers grown in Ham’s F12 (normalized intensity values) of the expression of WNT4 (A), Nanog (B), SOX2 (C), SOX9 (D) and MMP7 (E). ‘Day 0’ is representative of log phase A549 monolayers.
(TIF)

S4 Fig Box and whisker plots of microarray RNA gene expression in A549 monolayers grown in Ham’s F12 (normalized intensity values) of the expression of complement components C3 (A), C4b (B) and C5 (C). ‘Day 0’ is representative of log phase A549 monolayers.
(TIF)

S5 Fig Relative expression of surfactant protein genes by delta-delta Ct QRT PCR Taqman analysis of human primary ATII isolated from three separate donors. Donor 1 (chequered bars), Donor 2 (hatched bars) and donor 3 (speckled bars). ATII cells from Donor 2 were used for the RNA micro array analysis. ATP5B and TOP1 were used as reference genes. SFTPD, A1, B and C expression was relative to log phase A549 cells. SFTPA2 expression was relative to 25 day differentiated A549 cells.
(TIF)

S6 Fig Box and whisker plots of microarray gene expression in A549 monolayers grown in Ham’s F12 (normalized intensity values) of RNA expression ATP-binding cassette lipid transporters; ABCC11 (A), ABCC3 (B), ABCG1 (C), ABCA1 (D), ABCD4 (E), ABCC8 (F), ABCA12 (G), ABCB4 (H), ABCG2 (I), ABCC6P1 (J), ABCD3 (K) and ABCC6 (L). ‘Day 0’ is representative of log phase A549 monolayers.
(TIF)
