# Supplementary figures and images for: Long Term Culture of the A549 Cancer Cell Line Promotes Multilamellar Body Formation and Differentiation towards an Alveolar Type II Pneumocyte Phenotype
Source: PLoS One. 2016 Oct 28;11(10):e0164438. doi: 10.1371/journal.pone.0164438 (PMC5085087; doi:10.1371/journal.pone.0164438)

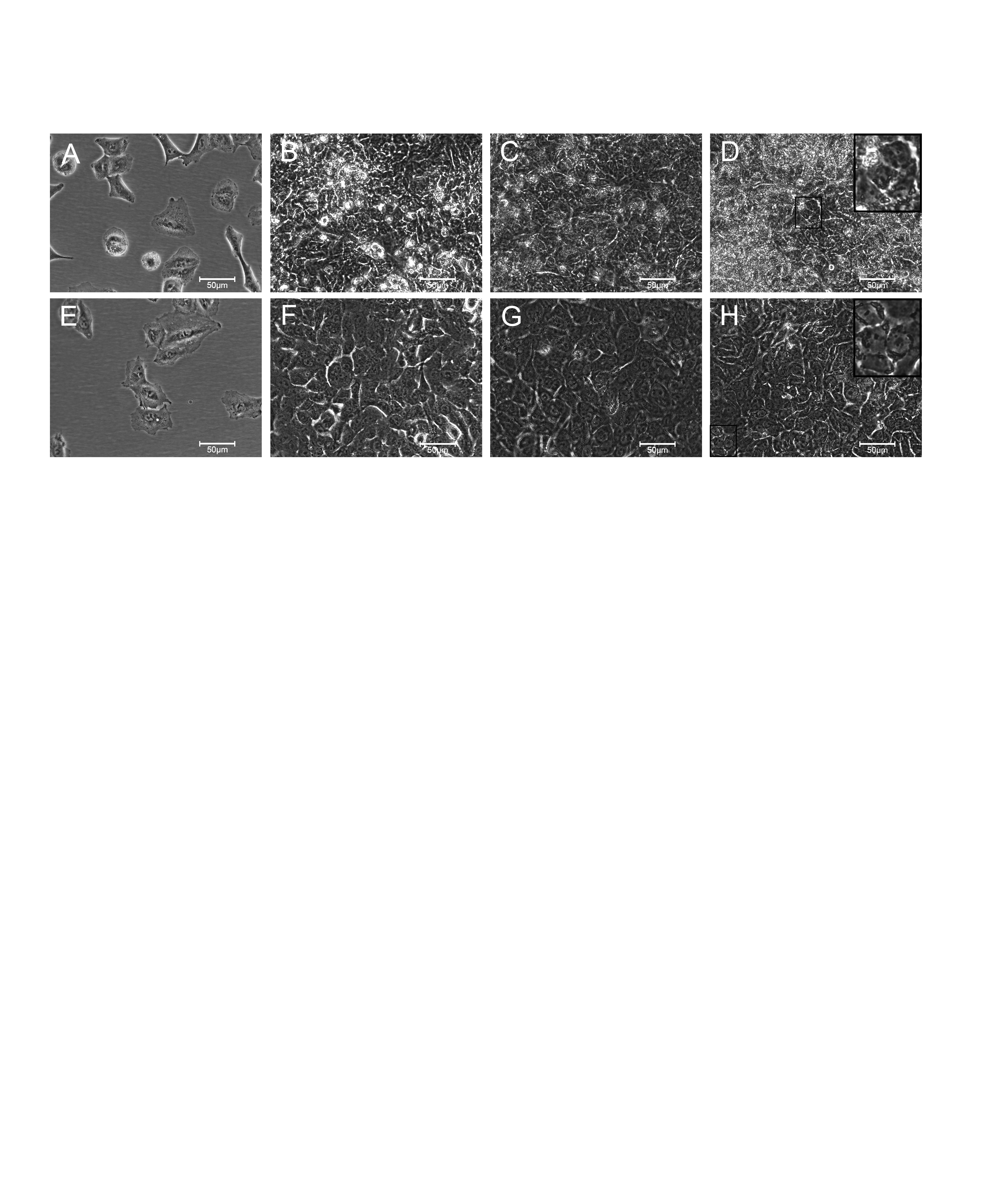

Supplement: S1 Fig — Photomicrographs show morphology at day 1 (A and E), days 8 (B and F) and 14 (C and G) and day 25 (D and H). (Inset in H shows higher magnification of cells displaying organized vesicles in F12, inset in D shows a higher magnification of cells grown in DMEM for comparison). (TIF) [file pone.0164438.s001.tif]

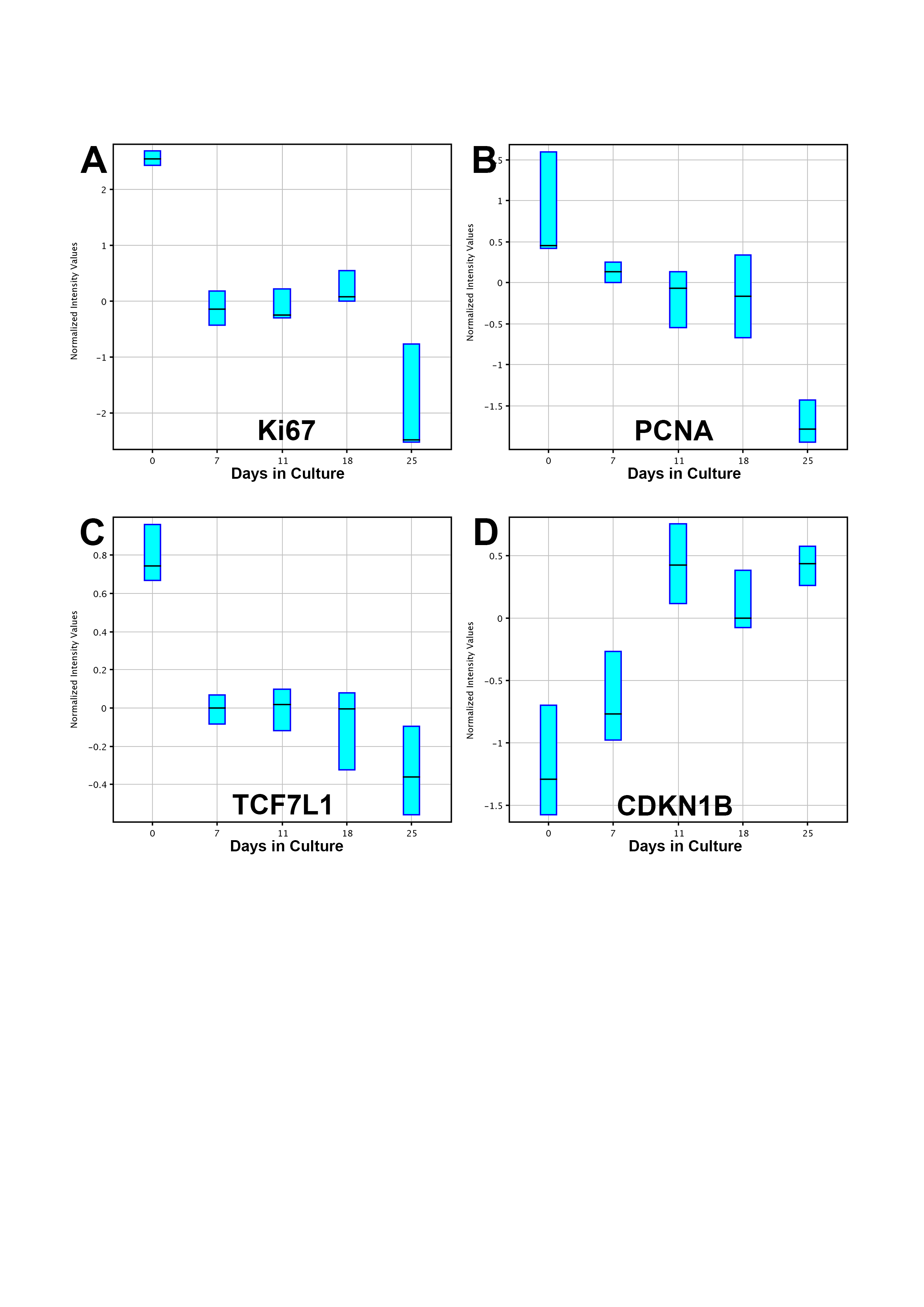

Supplement: S2 Fig — ‘Day 0’ is representative of log phase A549 monolayers. (TIF) [file pone.0164438.s002.tif]

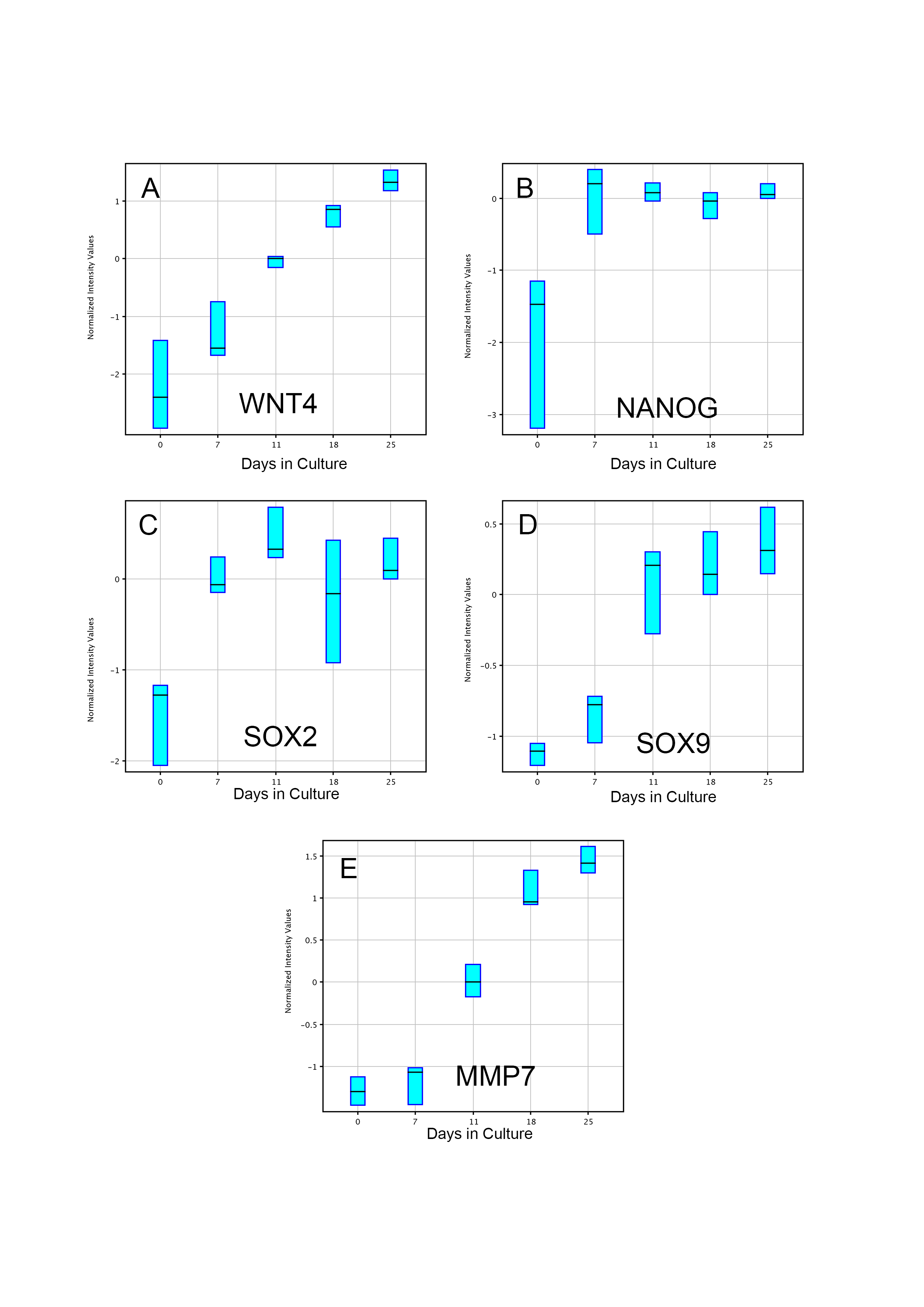

Supplement: S3 Fig — ‘Day 0’ is representative of log phase A549 monolayers. (TIF) [file pone.0164438.s003.tif]

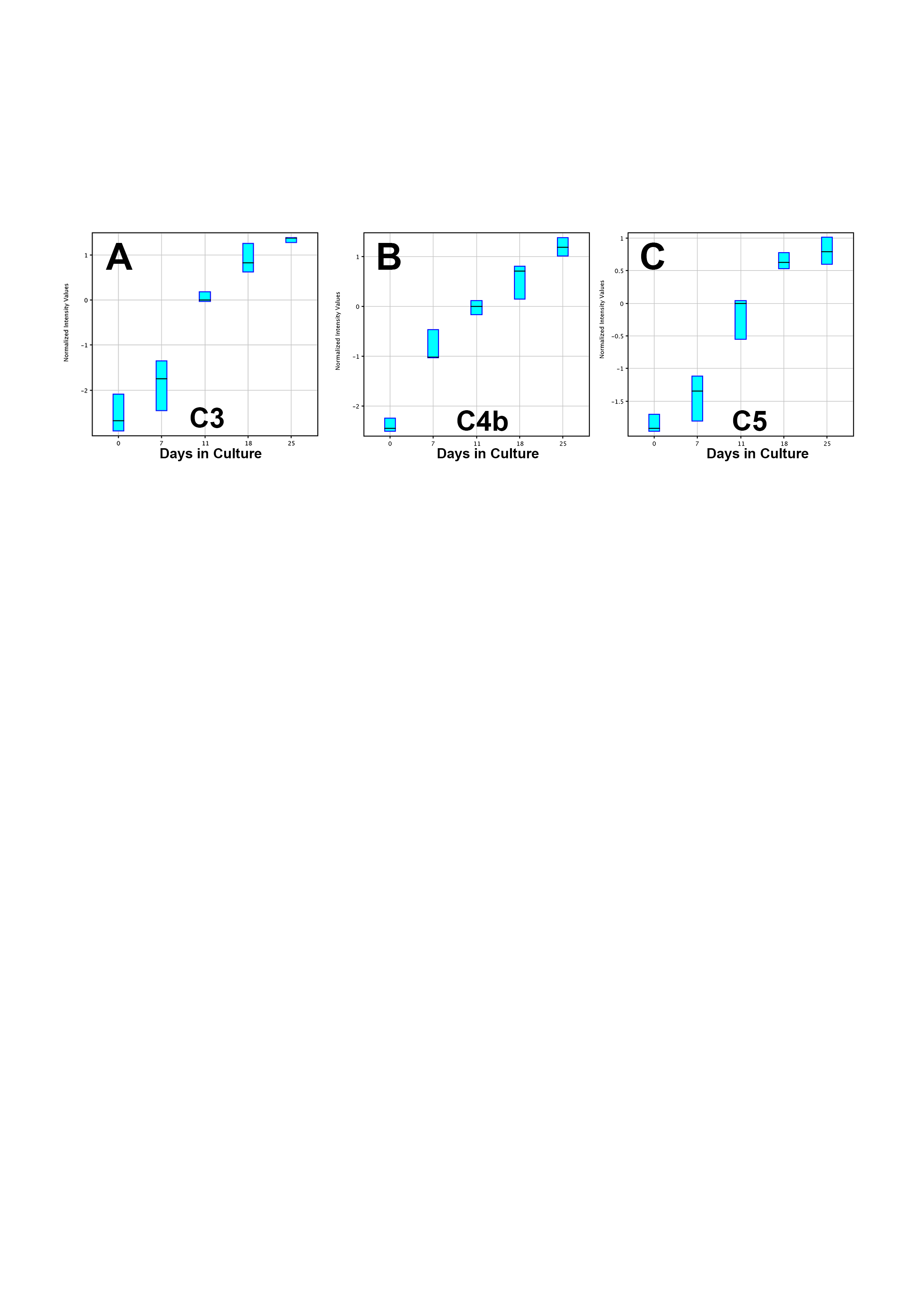

Supplement: S4 Fig — ‘Day 0’ is representative of log phase A549 monolayers. (TIF) [file pone.0164438.s004.tif]

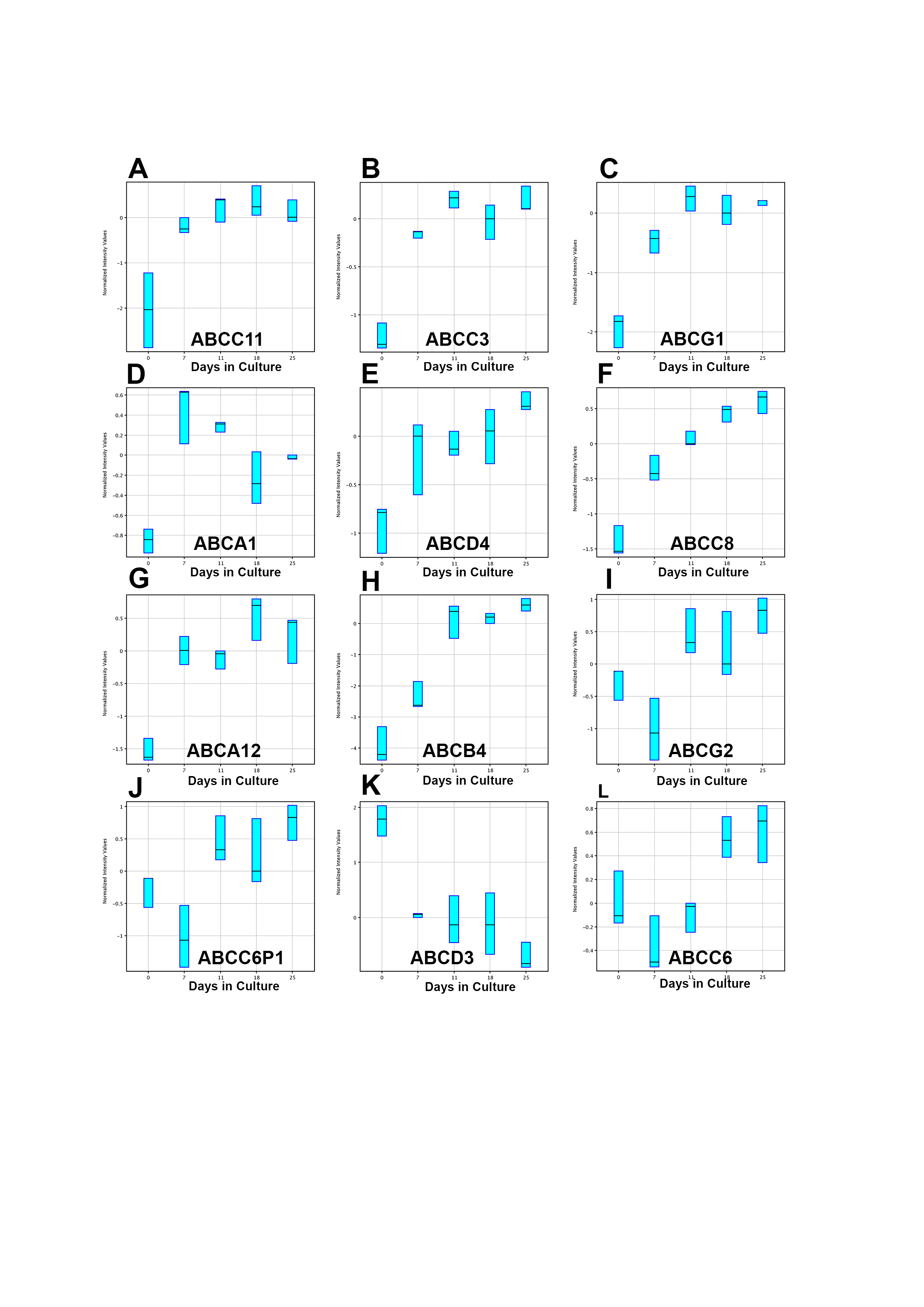

Supplement: S6 Fig — ‘Day 0’ is representative of log phase A549 monolayers. (TIF) [file pone.0164438.s006.tif]
